# Molecular Mechanisms Underlying the Beneficial Effects of Exercise on Brain Function and Neurological Disorders

**DOI:** 10.3390/ijms22084052

**Published:** 2021-04-14

**Authors:** Kévin Nay, William J. Smiles, Jacqueline Kaiser, Luke M. McAloon, Kim Loh, Sandra Galic, Jonathan S. Oakhill, Andrew L. Gundlach, John W. Scott

**Affiliations:** 1 St Vincent’s Institute of Medical Research, Fitzroy, Victoria 3065, Australia; kevin.nay@acu.edu.au (K.N.); wsmiles@svi.edu.au (W.J.S.); jkaiser@svi.edu.au (J.K.); lmcaloon@svi.edu.au (L.M.M.); kloh@svi.edu.au (K.L.); sgalic@svi.edu.au (S.G.); joakhill@svi.edu.au (J.S.O.); 2 Exercise and Nutrition Research Program, Mary MacKillop Institute for Health Research, Australian Catholic University, Melbourne, Victoria 3000, Australia; 3 Department of Medicine, University of Melbourne, Parkville, Victoria 3010, Australia; andrew.gundlach@florey.edu.au; 4 The Florey Institute of Neuroscience and Mental Health, Parkville, Victoria 3052, Australia

**Keywords:** exercise, brain, BDNF, iron, microbiota, Alzheimer’s disease, Parkinson’s disease

## Abstract

As life expectancy has increased, particularly in developed countries, due to medical advances and increased prosperity, age-related neurological diseases and mental health disorders have become more prevalent health issues, reducing the well-being and quality of life of sufferers and their families. In recent decades, due to reduced work-related levels of physical activity, and key research insights, prescribing adequate exercise has become an innovative strategy to prevent or delay the onset of these pathologies and has been demonstrated to have therapeutic benefits when used as a sole or combination treatment. Recent evidence suggests that the beneficial effects of exercise on the brain are related to several underlying mechanisms related to muscle–brain, liver–brain and gut–brain crosstalk. Therefore, this review aims to summarize the most relevant current knowledge of the impact of exercise on mood disorders and neurodegenerative diseases, and to highlight the established and potential underlying mechanisms involved in exercise–brain communication and their benefits for physiology and brain function.

## 1. Introduction

Both neurological and mental health disorders remain a significant medical challenge worldwide. It has been estimated that roughly 30% of the world’s population have experienced a mental disorder at some time in their life [1], while neurological disorders accounted for more than 9 million deaths in 2016 alone [2]. In 2018, a Lancet Commission report on mental health indicated that mental health disorders were on the rise in every country in the world and were predicted to cost the global economy $16 trillion by 2030 [3]. Moreover, the global cost associated with dementia alone was estimated to reach $948 billion during 2016 [4]. Therefore, the need for effective strategies to prevent, treat and improve neurological and mental health disorders is now critical to reduce this massive, global economic burden.

In this regard, physical activity (exercise) has become a progressively common and economically viable therapeutic strategy with positive effects on both neurological and mental health [5,6,7,8]. Exercise can generally be classified as one of two different types, endurance and resistance. Endurance exercise is classically performed against a relatively low load over a long duration, whereas resistance exercise is performed against a relatively high load for a short duration [9]. In general, individuals who train with endurance exercises develop better oxygen delivery and consumption within the skeletal muscle, whereas those who base their training on resistance exercises get bigger and stronger muscles [9]. However, most activities combine both endurance and resistance [9]. In this review, we outline the current knowledge of the impact of exercise on mood disorders and neurodegenerative disease, and highlight the potential underlying mechanisms involved in the organs-to-brain signalling axis in response to exercise.

## 2. Protective Role of Exercise in Preventing Mood Disorders and Neurodegenerative Diseases

Over three decades ago, Farmer et al. [10] reported that sedentary behaviour may be an independent risk factor contributing to the depressive symptoms experienced in a large cohort of adults. Indeed, exercise has been consistently described as an effective treatment for depression [11]. Several randomized controlled trials demonstrated that physical activity alone, or performed in combination with the administration of antidepressants, had a moderate-to-large effect on reducing depressive symptoms compared to physically inactive controls [12]. Interestingly, sensitivity to anxiety has also been inversely correlated with physical activity levels in healthy young adults [13]. In this regard, the positive impact of exercise on ameliorating symptoms associated with anxiety disorders (including general anxiety disorder, panic disorder, posttraumatic stress disorder, obsessive compulsive disorder, social anxiety disorder and specific phobias) has been well documented [14]. Aside from mood disturbances, exercise can also improve the symptomatology in individuals diagnosed with schizophrenia [15,16], autism [17,18] and bipolar disorder [19]. Based on these data, the current consensus is to advocate for increasing habitual levels of physical activity to improve mood state and mental health [20].

Exercise therapy is also known to have a direct, positive impact on neurodegenerative diseases and acquired brain injuries. Parkinson’s disease (PD) is a progressive nervous system disorder related to cell damage in the brain that affects motor control and cognitive function. Indeed, the main pathological characteristics of PD are cell death in the basal ganglia (affecting up to 70% of the dopaminergic neurons in the area of the brain known as the substantia nigra) and the presence of Lewy bodies (accumulations of the protein α-synuclein) in many of the remaining neurons [21]. The most obvious early symptoms are tremor, rigidity, slowness of movement, and difficulty walking [22]. Clinical studies have demonstrated that physical exercise improves symptoms in PD patients [23,24]. In animal models of PD, exercise improved motor function through preservation of nigrostriatal dopaminergic neurons, protection of mitochondria and suppression of the nigrostriatal formation of Lewy bodies [25,26,27].

Alzheimer’s disease (AD) is another irreversible, progressive neurodegenerative disease characterized by the degeneration and the destruction of neurons and synapses in the cerebral cortex and certain subcortical regions, predominantly causing memory loss, anxiety and confusion [28]. It is known that a sedentary lifestyle might lead to an earlier AD onset. Recent studies have observed that exercise interventions were capable of attenuating the symptoms of neurodegeneration experienced by AD patients, with comparable observations in rodent models of AD [29,30,31,32,33]. In addition, Liang et al. [34] have shown that physical activity reduces the levels of AD-associated biomarkers (e.g., Pittsburgh compound-B, tau, phosphorylated tau) in the cerebrospinal fluid of cognitively normal older adults

Huntington’s disease is an inherited disease that causes the progressive degeneration of nerve cells in the brain. Currently, there is no cure for this disease which has a broad impact on an individual’s functional ability that usually results in movement, cognitive and psychiatric disorders [35]. Here again, exercise training seems to be a safe and feasible treatment approach in patients with a possible positive effect on cognition and motor function in patients suffering from Huntington’s disease [36].

Multiple Sclerosis is a disease in which the immune system damages the protective covering of nerves, inducing nerve demyelination and neuronal dysfunction. The symptoms of this disease include vision loss, pain on eye, fatigue and impaired coordination [37]. Within the last decade, exercise has been described as a potential treatment for patients with Multiple Sclerosis. Resistance and endurance exercise and aquatic therapy were shown to have positive impacts across a variety of specific symptoms of multiple sclerosis. This leads to a perceived increase in quality of life without any negative effects [38].

Some more recent studies have also shown encouraging results regarding the impact of exercise in patients with acquired traumatic brain injuries [39,40,41] and in analogous animal models [42]. For example, Seo et al. [43] reported that treadmill exercise may be able to enhance the survival of cerebellar Purkinje neurons in traumatic brain injury-induced indirect cerebellar injury in rats. In mice, improved brain injury outcomes following voluntary exercise preconditioning were associated with an increase in the expression of specific neuroprotective genes and proteins (e.g., VEGF-A and EPO) in the brain [44]. A recent study using mice reported that 25 days of voluntary physical exercise initiated 11 days after controlled cortical impact injury reversed specific memory deficits associated with the traumatic brain injury [45].

Cerebrovascular diseases, including stroke, carotid-, vertebral- and intracranial stenosis, aneurysms and vascular malformations, are a range of conditions that affect the supply of blood to the brain. The resultant oxygen-deprivation damages brain cells in the affected area [46]. Interestingly, some recent clinical reports suggest that physical activity has a positive impact on stroke prevention and outcomes in cerebrovascular diseases [47,48]. These studies demonstrated that physically active people are more likely to have less severe strokes and to experience improved stroke outcomes compared to inactive people [47,48]. In rodents, voluntary training on running wheels or exercise on a treadmill reduced cerebral infarct size and functional deficits, improved endothelium-dependent vasorelaxation, and augmented cerebral blood flow [49]. Thus, exercise seems to represent a prophylactic treatment strategy for increasing blood flow and reducing brain injury caused by cerebrovascular diseases.

**Figure 1:**
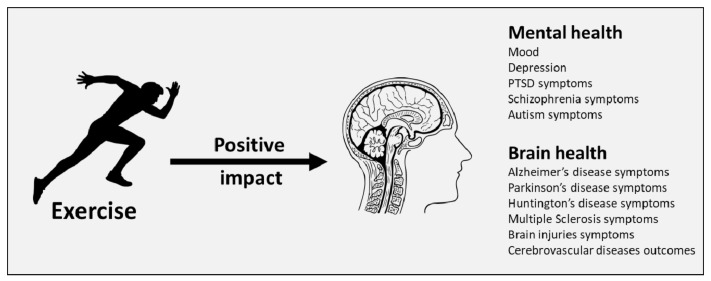
Impact of exercise on mental and brain health.

Together, these data indicate that exercise represents a highly efficacious intervention to prevent neurological disorders and associated psychological disturbances, by preserving mood and neuron integrity (Figure 1). Inclusion of exercise as an adjunct therapy alongside other treatments should greatly reduce mortality without the added burden of side-effects that can arise from pharmacological interventions.

## 3. BDNF: A Key Trophic Signalling Molecule in the Brain

Brain-derived neurotrophic factor (BDNF), or “abrineurin”, is a member of the neurotrophin family of growth factors related to the canonical, nerve-growth factor [50]. Within the neurotrophin family, BDNF exhibits the highest level of expression within the brain [50], where it binds to its primary receptor, tropomyosin receptor kinase B (TrkB), forming a BDNF-TrkB complex that becomes internalized and activates a plethora of signalling events involved in neuronal function [50]. TrkB is highly expressed in the hippocampus, a complex brain structure embedded in the temporal lobe of the human brain with a major role in learning and memory. The hippocampus is a highly plastic and vulnerable structure that is readily damaged by a variety of stimuli. It is the earliest and most severely affected structure in several neurodegenerative disorders [51]. High levels of BDNF in the brain are associated with improvements in memory and recollection, and the restraint of cognitive decline [52,53]. Conversely, decreased levels of BDNF are associated with a deterioration in memory function, neurodegeneration and cognitive impairments related to AD and PD [54,55]. In animal models, voluntary and forced running exercise has been linked to an increase in BDNF expression in the hippocampus [56,57]. Notably, when active rats were treated with a specific immunoadhesin chimera that mimics TrkB and consequently blocks BDNF in the hippocampus, they were refractory to the exercise-induced improvements in learning capacity, suggesting that BDNF is a key driver of these exercise-dependent benefits in brain [58]. More importantly, depending on the duration and intensity of the stimulus, exercise had a dose–response effect on BDNF expression, whereby the magnitude of this effect could be reinforced by habitual exercise bouts [59]. Studies in humans have shown that BDNF is released from the brain into the circulation during a single bout of endurance-based exercise and after 3 months of endurance training [60,61]. Similar results have also been detected by a meta-analysis of human studies that revealed that circulating BDNF was consistently increased after a session of exercise and that regular exercise intensified this effect [59]. It is worth noting that the magnitude of these effects may be lower in females when compared to males [59]. Indeed, across all studies, the authors found a significant negative correlation between effect size and percentage of women in the studies, and effect sizes were smaller for studies with a greater proportion of women [59]. This gender-dependent difference could be due to an interaction between sex hormones and BDNF, as observed in rodent models [62,63]. BDNF is generated in human skeletal muscle in response to exercise, but muscle-derived BDNF is not released into the circulation [64], suggesting that the links between skeletal muscle and BDNF-induced improvements in hippocampus in response to exercise are indirect.

## 4. Hormones and Metabolites of the Muscle–Brain Axis

Skeletal muscle is now described as a secretory organ [65], whereby in this context the term “myokine” is used to characterize cytokines and other peptides that are generated and/or expressed and released into the circulation by muscle fibers where they exert either autocrine, paracrine or endocrine effects [65]. Recent advances demonstrate that skeletal muscle produces myokines and metabolites in response to exercise, which permit crosstalk between the muscle and distal organs [66]. In this section, we describe the major myokines and metabolites identified as key players of the muscle–brain axis.

- **Irisin**

**Figure 2:**
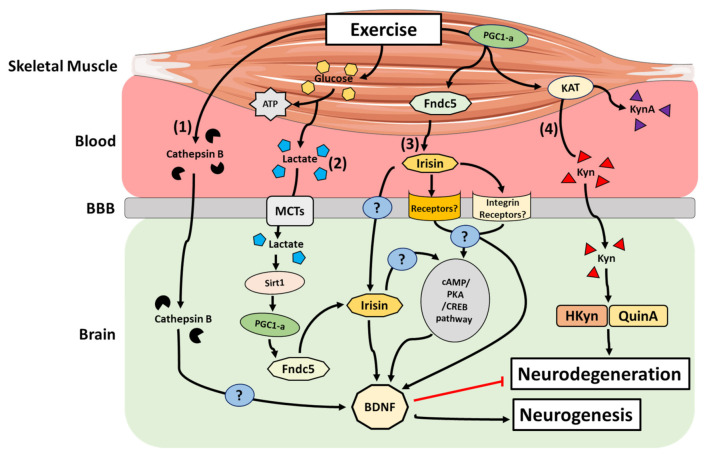
Main mechanisms by which active skeletal muscle can impact the brain. (1) Cathepsin B transcription is upregulated in brain and skeletal muscle, and its concentration increases in the blood in response to exercise. Cathepsin B is able to cross the BBB and may induce BDNF transcription in hippocampal neurons. (2) During exercise, glycolysis converts glucose into pyruvate to produce ATP. Lactate is produced from this reaction and accumulates in the blood. Lactate can cross the BBB and activate the PGC1α/FNDC5 signalling pathway and promote BDNF expression. (3) Exercise increases the release of irisin into the circulation. Irisin can cross the BBB and/or trigger several signalling pathways (including the cAMP/PKA/CREB) to activate BDNF production. (4) Kynurenine aminotransferases are increased in ‘trained’ skeletal muscles. These enzymes convert Kyn into kynurenic acid (KynA). Consequently, peripheral Kyn-to-KynA conversion is increased and prevents the accumulation of Kyn and its related neurotoxic metabolites (3-hydroxykynurenine or quinolinic acid) in the brain.

Irisin is a myokine encoded by the *FNDC5* gene and is involved in the browning of white adipose tissue, in turn increasing energy expenditure and improving glucose tolerance. The physiological benefits of irisin are illustrated by the fact that its circulating levels positively correlate with skeletal muscle mass and aerobic capacity [67,68]. Furthermore, acute and chronic exercise has been shown to increase the release of irisin into the blood [67,69]. Indeed, exercise induces an increase the expression and activity of muscle PGC-1α, which is accompanied by greater FNDC5 membrane expression. FNDC5 is cleaved, releasing irisin, which then enters the circulation [70]. Interestingly, irisin has been detected in brain tissue [71] and is associated with neuronal differentiation of mouse embryonic stem cells [72]. Peripheral delivery of FNDC5 to the liver via adenoviral vectors resulted in elevated blood irisin and induced expression of BDNF in the hippocampus, but not in the forebrain [73]. Furthermore, a recent study reported that after augmenting either brain or peripheral FNDC5/irisin levels, synaptic and memory impairments in rodent models of AD were attenuated [74]. These authors posited that FNDC5/irisin is likely a novel mediator of the beneficial effects of exercise on improving synapse function and memory in AD [74]. From a cell signalling perspective, endurance exercise stimulates increases hippocampal *FNDC5* gene expression through PGC-1α activation. This elevated *FNDC5* gene expression stimulates in turn *BDNF* gene expression [73]. Furthermore, other data revealed that irisin stimulates the cyclic AMP (cAMP)–protein kinase A (PKA)–cAMP responsive element-binding protein (CREB) pathway in cortical slices [74]. Interestingly, the PKA-cAMP-CREB pathway is known to be involved in the regulation of several genes including *BDNF* [75]. It remains unclear whether irisin induces these gene expression changes directly or indirectly through the action of a downstream effector [76]. A recent study identified integrin-αV as an irisin receptor in osteoclasts and adipocytes [77]. In parallel, integrin-αV has been detected in hippocampus and in prefrontal cortex in human [78]. These observations strongly suggest that integrin-αV could be a missing link in the understanding of irisin-BDNF interactions in response to exercise. However, the underlying mechanisms still remain unclear (Figure 2).

- **Lactate**

Lactate is an end-product metabolite of glycolysis and accumulates in skeletal muscle in response to contraction. Lactate can be used as an energy substrate by skeletal muscle and other tissues such as the heart and brain, or alternatively used to re-synthesize glucose in the liver via gluconeogenesis [79]. In the context of exercise, lactate can accumulate in the blood depending on the intensity and duration of the exercise stimulus [80]. From the bloodstream, lactate can effectively cross the blood–brain barrier (BBB), subsequently reaching neurons through the actions of monocarboxylate transporters (MCTs) [81]. Lactate is known to be a signalling molecule for neuronal plasticity in cultured mouse primary neurons and in vivo in the mouse sensory-motor cortex [82]. Recently, Lundquist et al. [83] observed an increase in the expression of BDNF in primary astrocytes treated with l-lactate in-vitro. They also demonstrated that l-lactate administration to healthy mice led to increased astrocyte morphological complexity, as well as astrocyte-specific neurotrophic expression within the striatum, a critical component of the motor and reward systems [83]. Interestingly, increases in peripheral blood lactate levels at rest in humans have been associated with an increase in circulating BDNF [84]. The underlying mechanism(s) linking lactate and BDNF levels remain obscure, but several hypotheses have emerged [85]; the most prominent hypothesis is based on the fact that during exercise, following its transport to the hippocampus via MCT-facilitated transport, lactate activates the PGC1α/FNDC5 signalling axis through activation of the Sirtuin 1 deacetylase (Sirt1) [86]. This activation in turn promotes BDNF expression, thus enhancing memory and learning (Figure 2) [86].

- **Cathepsin B**

Cathepsin B (CTSB) is a lysosomal cysteine protease of the papain family, which functions in intracellular protein catabolism and is implicated in various other physiological processes, such as processing of antigens in the immune response, hormone activation and bone turnover [87]. CTSB is ubiquitously expressed and upregulated in skeletal muscle after 11 weeks of strength training in healthy volunteers [88]. Moon et al. [89] demonstrated that following exercise in mice, *CTSB* transcription is upregulated in both brain and skeletal muscle, while its concentration is also known to increase in the circulation. The latter systemic increase after exercise was also observed in humans and non-human primates after a 4 month treadmill training intervention [89]. In the same study, the authors discovered that CTSB can cross the BBB and that exposure of hippocampal progenitor cells to exogenous CTSB can induce BDNF transcripts. In addition, CTSB knockout mice are resistant to the effects of voluntary exercise on hippocampal growth and resultant improvements in cognitive function [89]. Together, these studies suggest that exercise elevates systemic levels of CTSB, which, in turn, promotes the expression of hippocampal BDNF to stimulate neurogenesis (Figure 2).

- **Kynurenine**

Kynurenine (Kyn) is the first of many bioactive metabolites in the Kyn pathway shown to be involved in the modulation of the immune and central nervous systems (CNS) [90]. Importantly, Kyn is emerging as a crucial mediator of the muscle–brain axis in response to exercise (Figure 2) [91]. Indeed, alteration of Kyn pathway metabolism triggering an accumulation of the pathway metabolites, hydroxykynurenine (HKyn) and/or quinolinic acid (QuinA), is implicated in neurodegeneration, and thus, disease pathophysiology [92,93,94,95]. Notably, Kyn aminotransferases (KATs) are increased in habitually exercise-trained skeletal muscle [96,97], suggesting that these enzymes form part of the adaptive machinery responsive to exercise-induced metabolic stress. Specifically, KATs convert Kyn to kynurenic acid (KynA), which, unlike Kyn, does not efficiently cross the BBB. Agudelo et al. [96] have shown that exercise increases the expression of KATs through PGC-1α in both mouse and human skeletal muscle, which results in an increase in the peripheral Kyn-to-KynA conversion and thereby restrains the accumulation of Kyn and its downstream, neurotoxic metabolites in the brain. Notably, in mice, this detoxification mechanism was shown to reduce Kyn-induced excitotoxicity, neuroinflammation, and depressive-like behaviours [96].

## 5. Exercise and Liver–Brain Axis

Ketone bodies are water-soluble molecules derived from fatty acids and produced in the liver. Physical exercise induces the release of these compounds into the bloodstream, in particular β-hydroxybutyrate (β-HB) [98,99]. β-HB can be taken up in to the brain via the monocarboxylate transporters [100], and was previously recognised for its ability to confer neuroprotection in Alzheimer’s, Huntington’s and Parkinson’s disease [101,102,103]. Specifically, Sleiman et al. [104] demonstrated that β-HB can cross the BBB and increase BDNF transcription through the activation of BDNF promoters. Direct administration of β-HB onto neurons in vitro has been shown to increase BDNF expression [105,106].

In addition to β-HB, the liver has been shown to release the hepatokine, fibroblast-growth factor 21 (FGF21), in response to exercise [107,108]. In obese, insulin-resistant rats, subcutaneous injection of FGF21 prevented cognitive decline by improving hippocampal synaptic plasticity, dendritic spine density, brain mitochondrial function and cell apoptosis [109].

Finally, Insulin-like growth factor-1 (IGF-1) may also be a key player within the liver–brain axis in response to exercise. IGF-1, also referred to as somatomedin C, is a hormone similar in structure to insulin with important roles in childhood growth and anabolic effects in adults. IGF-1 is produced by several tissues, including the liver and skeletal muscle [110]. Although it remains unclear if skeletal muscle can secrete IGF-1 into the circulation [111], we know that the liver is the main source of IGF-1 that is released into the body [110], whereby 4 weeks of treadmill training in rats induced an increase in *IGF-1* mRNA levels in the liver [112]. Furthermore, undertaking resistance and high-intensity endurance-based exercise have been associated with increases in circulating levels of IGF-1 in humans [113,114]. In response to exercise, circulating IGF-1 has been shown to infiltrate the brain through the blood cerebrospinal fluid pathway, resulting in an increase in the number of new neurons, especially in the hippocampus [115,116]. Chen et al. [117] found that the effect of physical exercise on the upregulation of hippocampal BDNF expression and adult neurogenesis was blocked by neutralizing IGF-1 antibodies. Collectively, these studies suggest that IGF-1 is a major adaptive player in driving the liver–brain signalling axis in response to exercise.

## 6. Exercise and the Microbiome–Gut–Brain Axis

**Figure 3:**
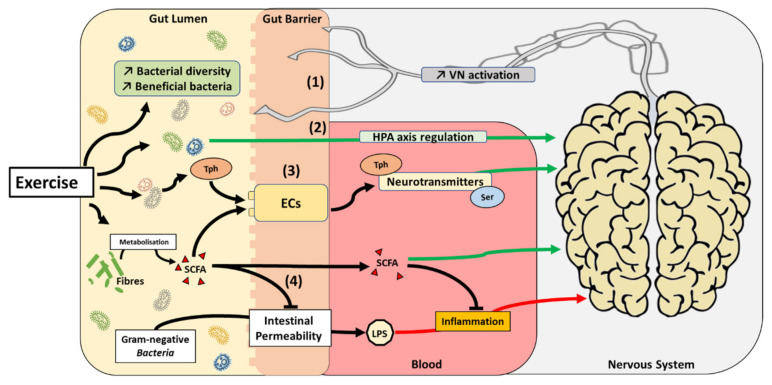
Proposed mechanisms by which exercise can impact the gut microbiota–brain axis. Exercise promotes the development of beneficial bacteria species and increase bacterial diversity in the gut. This phenomenon may have several impacts on the gut microbiota–brain axis: (1) activation of the vagus nerve (VN), (2) activation of the HPA axis, (3) modulation of neurotransmitter metabolism (i.e., tryptophane (Tph) and serotonin (Ser), and/or (4) reduction of inflammation by SCFA production.

The adult human gut contains over 10^14^ bacteria, which is equivalent to the number of somatic cells present in the human body. This complex ecosystem referred to as the gut microbiota has been described as a novel organ with hitherto undefined capabilities of communicating with distant organs in the body [118,119,120]. Gut microbes are able to communicate with the CNS, forming a microbiome–gut–brain axis [121]. This crosstalk can be altered by metabolic changes within this bacterial ecosystem, whereby physical activity can influence the relative abundance of butyrate producing taxa in the gut [122,123,124]. In humans, several studies have described a positive correlation between the level of fitness of an individual and the diversity of their microbiome [125,126,127]. However, in such populations, it remains challenging to control the exercise-induced diet alterations which can affect the gut microbiota independently of exercise [126]. Interestingly, physical exercise-induced behavioural and mental health improvements are associated with the alteration of specific strains of bacteria within the gut [123,128,129]. Intriguingly, transplantation of microbiota of depressed patients to rodents induces depressive-like behaviour [130]. Kelly et al. [131] also observed depressive behaviour and anxiety in rats in response to transplantation of fecal microbiota from depressed patients. While the precise, underlying mechanisms of this communication between the gut microbiota and the brain in response to exercise is poorly-defined, several hypotheses have been proposed (Figure 3) [132].

The vagus nerve (VN) is the only cranial nerve that exits the brain, connecting the CNS to several other organs, including the gut. Activation of the VN is associated with an improvement of depressive symptoms in diabetic rats [133]. Interestingly, a surgical operation in which one or more branches of the VN are cut (vagotomy) abrogates the effects of probiotic supplementation in preventing mood disorders in different rodent models reproducing anxiety and depression [134,135,136]. However, empirical evidence is still lacking to unequivocally determine the influence exercise may have on the diversity of gut flora and how this could impact the gut–brain axis via the VN.

The hypothalamic–pituitary–adrenal (HPA) axis is an interactive neuroendocrine unit comprised of the hypothalamus, the pituitary gland, and the adrenal gland that regulates physiological responses to a multitude of stressors [137]. It has been suggested that commensal microbiota can affect the postnatal development of the HPA axis stress response in mice [138]. Interestingly, modulation of gut microbiota with probiotics is associated with an improvement of HPA axis dysfunction and improved cognitive function and mental health [139,140,141]. As exercise can impact HPA axis [142,143], these data suggest that by modulating gut microbiota, exercise could play a role in HPA axis regulation.

Short-chain fatty acids (SCFAs) contain fewer than six carbon atoms and are produced in the colon by bacterial fermentation of dietary fiber and resistant starch [144]. Even if only a minor fraction of colon-derived SCFAs reaches the systemic circulation and other tissues, they can readily cross the BBB and have neuroactive properties. However, the precise mechanisms involved in the action of SCFAs on the CNS remain largely unknown [145]. As mentioned, exercise increases the relative abundance of taxa-producing SCFAs, in particular butyrate [127,146,147]. SCFAs seem to play an important role in maintaining the integrity of the BBB, which is closely associated with the controlled passage of molecules and nutrients from the circulation to the brain, playing a central role in brain development and the preservation of CNS homeostasis [148]. Systemic injection of butyrate has been reported to induce histone hyperacetylation in the hippocampus and the frontal cortex, accompanied by antidepressant-like effects and increased levels of *BDNF* transcripts in the brain [149]. Other laboratories have shown that butyrate stimulates neural proliferation in the dentate gyrus in mice and butyrate has also been used to induce neurogenesis after ischemic brain insult in adult rodents [150,151]. Furthermore, it is now known that increased gastrointestinal permeability is related to the translocation of lipopolysaccharide (LPS) from Gram-negative bacteria, a phenomenon potentially playing a role in exacerbating mood disorders and neurodegeneration [152,153]. Interestingly, exercise is associated with a greater abundance of commensal bacterial populations that reduce intestinal permeability, limiting the release of LPS and consequent systemic increases in inflammatory markers [154]. Mechanistically, butyrate reduces gut permeability by augmenting mucus production and modulating synthesis of tight-junction proteins [155]. This phenomenon in turn may limit the accumulation of LPS in the blood, consequently decreasing endotoxemia-related inflammation [156]. Moreover, SCFAs act on leukocytes and endothelial cells through at least two mechanisms: activation of free fatty acid receptors type 2 and 3; and inhibition of histone deacetylases, regulating several leukocyte functions including production of pro-inflammatory cytokines. Therefore, the neuroprotective and anti-inflammatory properties of SCFAs indicate a prominent role within the gut–brain axis.

Finally, the gut microbiota is now known to play a role in neurotransmitter metabolism, particularly of serotonin and tryptophan [157,158]. The vast majority of serotonin (>90%) in the human body is produced by enterochromaffin (EC) cells of the gut, where L-tryptophan is converted by tryptophan hydroxylase 1 to serotonin [159]. When released into the bloodstream, serotonin improves mood state by preventing anxiety and depression [160]. Indeed, serotonin is a neurotransmitter throughout the body acting via 14 different serotonin receptor types to produce diverse effects on mood, anxiety, sleep, appetite, temperature, eating and sexual behaviour, movement, and gastrointestinal motility [161]. Germ-free (GF) mice that are laboratory-raised and gut microbiota-deficient display increased levels of circulating tryptophan [162] and decreased serotonin [163]. Colonisation of GF mice decreases circulating levels of tryptophan and improves their anxiety state [162]. Furthermore, autistic-like mice displayed improvements in their behaviour when supplemented with the bacterial strain, *Bacteroides fragilis*, which is involved in tryptophan metabolism [157]. Finally, it has been demonstrated that some indigenous bacterial strains in the gut promote serotonin biosynthesis and release from the enterochromaffin cells (ECs) via the actions of SCFAs [164]. Known to promote SCFA producing strains in the gut microbiota [127,146,147], exercise may impact the gut–brain axis by altering neurotransmitter metabolism.

Together, these data suggest that the gut microbiota could be a major contributor to the benefits for brain function observed in response to exercise, but currently, the underlying mechanisms remain unclear. Further research is required to better understand if and how exercise influences the microbiome, and the mediators involved in the gut–brain axis.

## 7. Iron: An Emerging Factor in the Muscle–Brain Axis

Iron is a key element that can cross the BBB to mediate multiple biological processes in the brain, including the transmission of the respiratory chain, generation of neurotransmitters, and axonal myelination [165]. Hepcidin, a 25-amino-acid peptide hormone secreted by the liver, is the main regulator of iron metabolism via its inhibition of iron export by degradation of the iron exporter, ferroportin [166]. In addition, hepcidin reduces iron absorption by duodenal enterocytes. Consequently, hepcidin induces a decrease in the availability of iron in the blood and an increase in iron storage in cells that express ferroportin [166]. As iron turnover is very slow and there is no physiological regulatory mechanism for excreting it, iron can accumulate in brain and trigger an age-dependent development of the pathophysiology of diseases such as PD and AD, via oxidative stress-induced damage [167]. Indeed, ferritin, a protein that stores iron, has been ascribed a role in neurodegeneration processes [168]. Furthermore, iron deficiency and/or overload alter emotional behavior and drive anxiety [169]. Notably, recent studies in rodent models of physical hypoactivity have reported an increase in hepcidin levels and a disruption of iron homeostasis characterized by an increased storage of iron in organs including spleen, liver and bone [170,171,172]. Although the organs impacted differed depending on the duration of inactivity, these results in rodents were consistent with a study in humans, which demonstrated that 5 days of hypoactivity increased hepcidin levels and splenic iron content [173]. It has been suggested that this hepatic release of hepcidin in the early stage of hypoactivity was mainly triggered by inflammation [171,173,174]. These data indicate that physical activity status can modulate iron storage in the brain through regulation of hepcidin levels. Unfortunately, brain iron content was not measured in these studies [170,171,172,173]. Paradoxically, in humans, physical exercise also induces an upregulation of hepcidin levels in response to inflammatory processes mainly due to muscle interleukin-6 release [175,176]. These observations suggest exercise could promote iron accumulation in the brain in a similar way as physical inactivity. However, a recent study revealed that brain tissue iron levels were significantly increased in AD mice, but a treadmill exercise regimen reestablished levels similar to those in non-transgenic control mice [177]. These authors also suggest that exercise reduces AD-induced cognitive decline and neuronal cell death by lowering iron levels in the brain [177]. Altogether, these data strongly support the idea that iron has a role in the muscle–brain axis during physical activity and that iron-targeted therapeutic strategies involving exercise might be useful for patients with neurodegenerative disease and emotional behavior disorders.

## 8. Conclusions and Future Directions

The ability of exercise to produce beneficial effects on, and maintain, physical and mental health are established. However, the underlying mechanisms responsible for these beneficial effects remain poorly defined. Indeed, although some myokines and metabolites released by skeletal muscle are known to positively impact brain health, the underlying mechanisms require further investigation. In addition, important questions remain about new targets such as gut microbiota and iron metabolism and their roles in the muscle–brain axis in response to exercise. Such recent advances in the field may facilitate the development of new and improved interventions to optimize mental and brain health. Mimicking and/or increasing the positive actions of physical exercise on the brain through the identification of new drugs or other physiological interventions would be a major step towards enhancing the mental and health wellbeing of all members of our local and global societies.

## Data Availability

Not applicable.

## Abbreviations

AD	Alzheimer’s disease
BBB	Blood–brain barrier
BDNF	brain-derived neurotrophic factor
cAMP	cyclic adenosine monophosphate
CNS	central nervous system
CREB	cAMP-response element-binding protein
CTSB	cathepsin B
EPO	erythropoietin
FGF21	fibroblast growth factor 21
FNDC5	fibronectin type III domain-containing Protein 5
HKyn	hydroxykynurenine
HPA	hypothalamic-pituitary-adrenal
IGF-1	insulin-like growth factor 1
KATs	kynurenine aminotransferases
KPM	kynurenine pathway metabolism
Kyn	kynurenine
LPS	lipopolysaccharide
MCTs	monocarboxylate transporters
PD	Parkinson’s disease
PGC1α	peroxisome proliferator-activated receptor gamma coactivator 1-alpha
PKA	protein kinase A
QuinA	quinolinic acid
SCFAs	short chain fatty acids
Ser	serotonin
SIRT1	sirtuin 1
Tph	Tryptophane
TrkB	tropomyosin receptor kinase B
VEGF	vascular endothelial growth factor
VN	vagus nerve
β-HB	β-hydroxybutyrate
